# Surgical and perioperative management of flail chest with titanium plates: a French cohort series from a thoracic referral center

**DOI:** 10.1186/s13019-023-02121-8

**Published:** 2023-01-18

**Authors:** Sarah Féray, Clarisse Blayau, Hicham Masmoudi, Samuel Haddad, Christophe Quesnel, Jalal Assouad, Muriel Fartoukh

**Affiliations:** 1 Assistance Publique-Hôpitaux de Paris, Hôpital Tenon, Service de Médecine Intensive Réanimation, Sorbonne Université, 75020 Paris, France; 2 Assistance Publique-Hôpitaux de Paris, Hôpital Tenon, Service d’Anesthésie-Réanimation et Médecine Péri-operatoire, Sorbonne Université, 75020 Paris, France; 3 Assistance Publique-Hôpitaux de Paris, Hôpital Tenon, Service de Chirurgie Thoracique et Vasculaire, Sorbonne Université, 75020 Paris, France; 4 Assistance Publique-Hôpitaux de Paris, Hôpital Tenon, Service de Radiologie, Sorbonne Université, 75020 Paris, France

**Keywords:** Thoracic trauma, Flail chest, Surgical rib fracture fixation, Complication, TTSS score

## Abstract

**Background:**

The development of titanium claw plates has made rib osteosynthesis easy to achieve and led to a renewed interest for this surgery. We report the management of patients referred to the intensive care unit (ICU) of a referral center for surgical rib fracture fixation (SRFF) after chest trauma.

**Methods:**

We performed a retrospective observational cohort study describing the patients’ characteristics and analyzing the determinants of postoperative complications.

**Results:**

From November 2013 to December 2016, 42 patients were referred to our center for SRFF: 12 patients (29%) had acute respiratory failure, 6 of whom received invasive mechanical ventilation. The Thoracic Trauma Severity Score (TTSS) was 11.0 [9–12], with 7 [5–9] broken ribs and a flail chest in 92% of cases. A postoperative complication occurred in 18 patients (43%). Five patients developed ARDS (12%). Postoperative pneumonia occurred in 11 patients (26%). Two patients died in the ICU. In multivariable analysis, the Thoracic Trauma Severity Score (TTSS) (OR = 1.89; CI 95% 1.12–3.17; *p* = 0.016) and the Simplified Acute Physiology Score II without age (OR = 1.17; CI 95% 1.02–1.34; *p* = 0.024) were independently associated with the occurrence of a postoperative complication.

**Conclusion:**

The TTSS score appears to be accurate for determining thoracic trauma severity. Short and long-term benefit of Surgical Rib Fracture Fixation should be assessed, particularly in non-mechanically ventilated patients.

**Supplementary Information:**

The online version contains supplementary material available at 10.1186/s13019-023-02121-8.

## Introduction

Chest trauma accounts for one-third of all trauma cases and may lead to fatal complications [1]. Flail chest, defined as 3 or more broken ribs in at least 2 sites, is a severe thoracic injury associated with chest wall instability and significant morbidity and mortality [2]. Patients may develop severe acute respiratory failure requiring mechanical ventilation and prolonged intensive care unit (ICU) and hospital stays. Long term morbidity may include chest deformity, chronic pain, disability and lung function compromise [3]. Age, pre-existing respiratory or cardiac disease, number of fractured ribs and pulmonary contusion [4–6] are recognized factors of severity. The Thoracic Trauma Severity Score (TTSS) has been developed for predicting the occurrence of acute respiratory distress syndrome (ARDS) and mortality in patients with chest trauma [7] (Additional file 1: Table S1). The Injury Severity Score (ISS) is an established medical score to assess trauma severity, and is associated with morbidity, length of hospital stay, and mortality [8].

The usual management of flail chest is based on measures including oxygen therapy, multimodal analgesia, respiratory physiotherapy and early rehabilitation [9–11]. The generalization of non-invasive ventilation has decreased the need for tracheal intubation and invasive ventilation, and its complications [12]. Despite some promising prospective trials, the surgical fixation of rib fractures (SRFF) is still debated. French referentials [13] recommend “surgical fixation in patients with flail chest and requiring mechanical ventilation if the respiratory status does not permit the weaning of mechanical ventilation within 36 h of admission”, on the basis of three small-size randomized controlled trials using different primary end points [12–14]. The development of titanium claw plates of the Stracos™ system (Strasbourg Costal Osteosynthesis System, MedXpert GmbH, Germany) makes easy to achieve rib fixation. This new technology has led to a renewed interest for the operative management approach and has led surgeons to consider expanding its indications.

We report our experience of the ICU management of thoracic trauma patients after SRFF referred to Tenon hospital, a University teaching hospital and referral thoracic center in Paris, France. The aim of this retrospective cohort study was to describe the characteristics of the patients, the surgical procedure, and the in-hospital outcomes, and to identify factors associated with the occurrence of postoperative complications.

## Methods

### Admission

From November 2013 to December 2016, all the consecutive patients admitted to the ICU of Tenon hospital for the perioperative management of chest trauma were eligible. Written informed consent was not required because of the retrospective nature of the investigation, which was approved by the Institutional Review Board of the French learned society for respiratory medicine - Société de Pneumologie de Langue Française (CEPRO 2019-010). Demographics and medical history were collected from the computerized medical records, Respiratory failure was defined as the need for high flow nasal oxygen (HFNO), non-invasive ventilation (NIV), or intubation with invasive ventilation. The TTSS was calculated on ICU admission, including the age of patients, the PaO2/FiO2 ratio, the number of fractured ribs, and the existence of pulmonary contusion and pleural involvement on computed tomography (CT)-scan. All patients had a radiological diagnostic of chest trauma. An expert radiologist from our center reviewed all CT scans in order to describe all lesions accurately.

### Surgery

The indications for surgery were the following: anterior and anterolateral flail chest (radiological), displaced rib fractures (more than 2 cm measured on CT scan), fractures impaling the lung or threatening other organs, and rib fractures causing pain refractory to medical treatment, including paravertebral bloc or epidural analgesia. Patients with severe head injury were contra-indicated for surgery. Surgery was postponed if the patient could not be installed on the lateral decubitus position or because of associated injuries (orthopedic or spinal trauma) whose treatment was more urgent.

The surgical approach was a posterolateral thoracotomy. An initial exploration of the cavity was performed, and associated with hemothorax drainage, if necessary. A bubble test was performed to look for any parenchymal air leak. The parenchyma was then sutured.

Osteosynthesis of the ribs of the flail chest or of the most displaced ribs was performed by placing the Stracos plates. The remaining broken ribs were fixed with wire to the ribs repaired with the plate. Once the reduction was considered satisfactory, two anterior and posterior endothoracic tubes were placed. After closing, the tubes were put under suction at − 20 cmH20.

The characteristics of the surgical procedure were collected: surgical approach, duration of surgery, perioperative transfusion and fluid intake, duration of lung exclusion and type of regional analgesia technique.

### Postoperative period

All patients received the same enhanced recovery after surgery protocol from our center (detailed in the Additional file 2), including early mobilization, early oral feeding, daily respiratory physical therapy, and pain control. Pain control was provided by multimodal analgesia including regional analgesia: all patients received a paravertebral catheter, except if the trauma involved more than 6 levels or if the trauma was bilateral in which case an epidural catheter was placed.

Postoperative respiratory failure was defined as a failure of extubation within the first postoperative 24 h, or as the need for HFNO, NIV or invasive ventilation. Postoperative hemodynamic failure was defined as the need for the administration of vasoactive drugs. Postoperative acute renal failure was defined as the need for renal replacement therapy. Postoperative pneumonia was defined by a clinical suspicion of pneumonia associated with a microbiological documentation. Lengths of ICU and hospital stay and respective vital status at discharge were reported.

### Statistical analysis

The primary endpoint was the rate of occurrence of postoperative complications, defined as at least one postoperative event among acute respiratory, hemodynamic or renal failure, postoperative pneumonia, surgical site infection or ICU death. The secondary endpoints were the factors associated with the occurrence of postoperative complications. Continuous variables are expressed as median and interquartile [25–75], and categorical variables as numbers (percentage). Continuous variables were compared using the Student's *t*-test or the Mann–Whitney test, according to their parametric distribution. Categorical variables were compared by a chi2 test or a Fischer test. The predictors of the occurrence of postoperative complications were assessed by univariate and multivariable analyses. The first part of the analysis measured the crude associations between variables of interest and postoperative complications, using Odds Ratio (OR) and their corresponding 95% confidence interval (95% CI). The multivariable analysis was performed by a step-by-step logistic regression model, integrating the selected variables in univariate analysis with a *p*-value < 0.1, and respecting the ratio of 1–10 events per variable. The area under the receiver operating characteristic (ROC) curve [33] was used to assess the discrimination of the model. Statistics were performed using Stata/tm 13.1 software (StataCorp, College Station, Texas, USA).

## Results

**Fig. 1:**
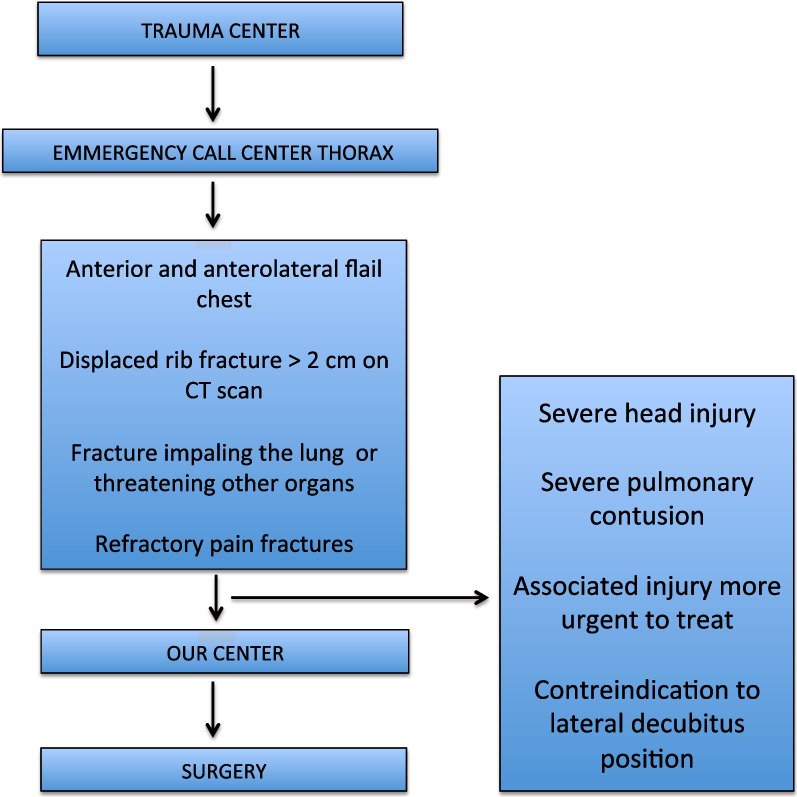
Diagram of the patient's course

**Fig. 2:**
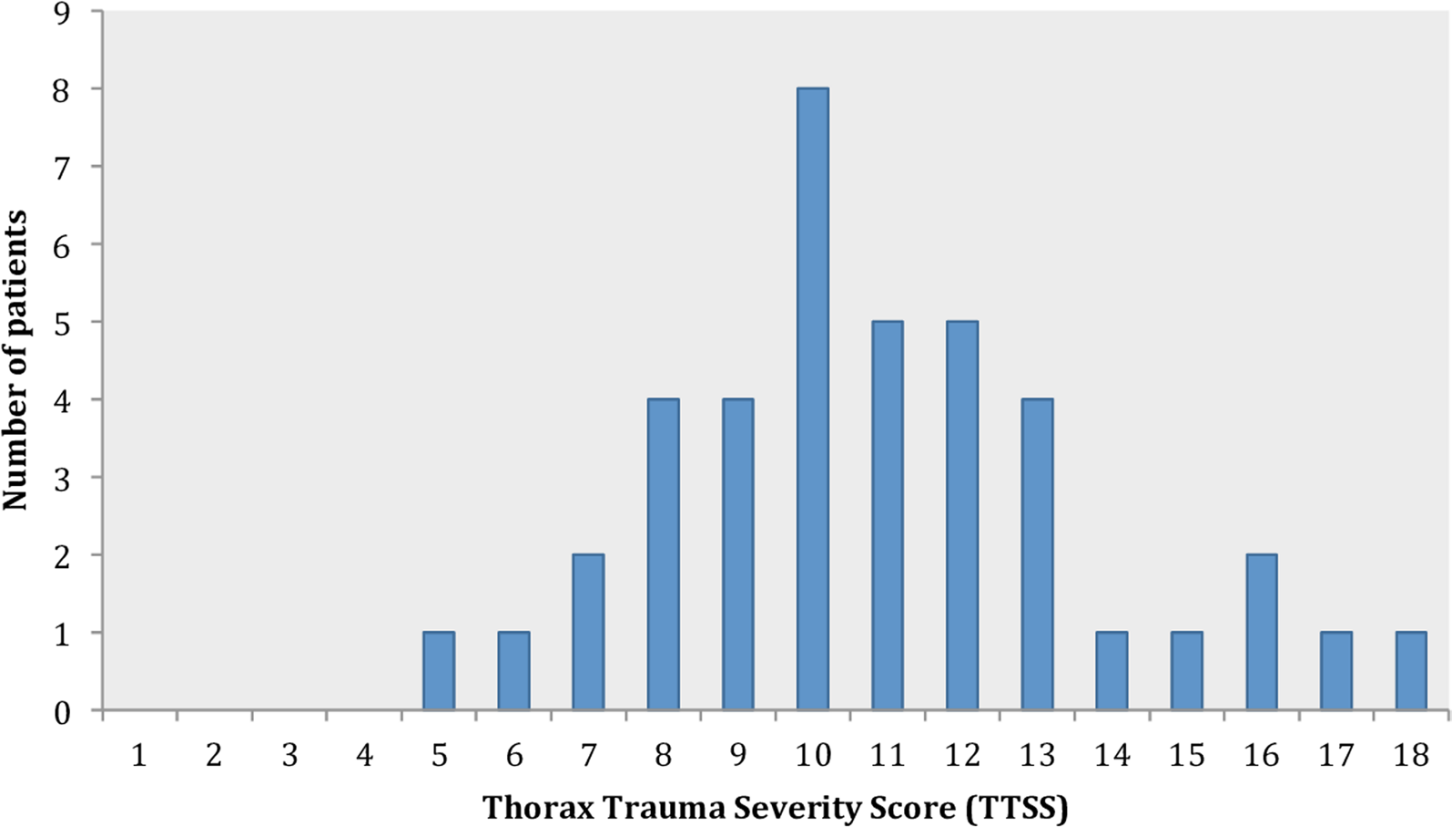
Distribution of the TTSS

During the study period, 42 patients were referred to our ICU from the trauma centers of Paris area (either Emergency Departments or ICUs), for the perioperative management of chest trauma after a median duration of 3.0 days [2.0–5.75]. The patient course is described in Fig. 1. The patients were 58-year old [46.5–77] with few comorbidities (Table 1). On preoperative assessment, 12 patients (29%) had acute respiratory failure, 6 of them required NIV and 6 required invasive ventilation. The mean duration of ventilation (invasive and non invasive) before surgery was 1 day [1–4]. The TTSS was 11.0 [9–12] (Fig. 2), with 7 broken ribs [5–8.75]. Pulmonary contusion was frequent (27/40, 67%). A flail chest was present in 39 patients (93%). The remaining three patients had 5 broken ribs (n = 2), and 8 broken ribs associated with a sternal fracture (n = 1). Associated extra thoracic injuries are described in the Appendix (Additional file 1: Table S2).

**Table 1:** Pre-operative characteristics

Variable	Value
Age (years), median [IQR 25–75]	58.3 [46.5–77.0]
Men, n (%)	28 (67)
SAPS II, median [IQR 25–75]	26 [18–34]
ISS, median [IQR 25–75]	21 [16–28]
TTSS, median [IQR 25–75]	11 [9–12]
Comorbidity	
Charlson score, median [IQR 25–75]	0 [0–1]
Smoking, n (%)	17 (40)
Cause of thoracic trauma, n (%)	
Road traffic accident	20 (48)
Domestic fall	17 (40)
Time from trauma to ICU admission (days), median [IQR 25–75]	3.0 [2–5.8]
Number of broken ribs, median [IQR 25–75]	7 [5–8.8]
Flail chest, n (%)	39 (93)
Pulmonary contusion, n (%)^(1)^	27 (67)
1 lobe	16 (40)
2 lobes	7 (17)
> 2 lobes	4 (10)
Pleural involvement, n (%)^(1)^	34 (85)
Isolated pleural effusion	3 (7)
Pneumothorax/Hemopneumothorax unilateral	29 (72)
Pneumothorax/Hemopneumothorax bilateral	1 (2)
Tension pneumothorax	1 (2)
Associated intra-thoracic fractures, n (%)^(2)^	20 (51)
Sternum	3 (8)
Clavicle	5 (13)
Scapula	9 (23)
Vertebrae	3 (8)
Extra-thoracic injury, n (%)	17 (40)
Head and neck injury, n	2
(Specific intervention needed), n	(0)
Face, n	4
(Surgery needed), n	(2)
Extremities, n	5
(Surgery needed), n	(3)
Abdomen, n	3
(Specific intervention needed), n	(1)
Spine, n	3
(Surgical or radiological procedures needed), n	(2)
Preoperative respiratory status, n (%)	
Respiratory failure *	12 (29)
Invasive ventilation	6 (14)
Non-invasive ventilation	6 (14)
High flow nasal oxygen therapy	1 (2)

SAPS II, Simplified Acute Physiologic Score; ISS, Injury Severity Score; ICU, intensive care unit; TTSS, Thoracic Trauma Severity Score

*NIV was used for hypoxemic and hypercapnic patients, while HFNO was administered to hypoxemic patients without hypercapnia. Without rapid clinical or biological improvement, patients received invasive mechanical ventilation

Date available for 40 patients ^(1)^ and 39 patients ^(2)^

SRFF was performed 4 days [2–6] after the trauma. The characteristics of intraoperative anesthesia are detailed in Table 2. Intraoperative ventilation difficulties occurred in 13 patients. A thoracic paravertebral catheter was inserted at the end of the surgical procedure in most patients (n = 31; 77%).

**Table 2:** Operative characteristics

Variable	Value
Time from trauma to surgery (days), median [IQR 25–75]	4 [2–6]
Red blood cells transfusion, n (%)	6 (14)
Vasopressor support (norepinephrine), n (%)^(1)^	11 (27)
Crystalloid fluid administration (ml/kg/h)^(2)^	7.21 [4.4–10.4]
Ventilation	
Selective intubation, n (%)	42 (100)
Ventilation difficulties, n (%)^(3)^	13 (31)
Duration of surgery (hours), median [IQR 25–75]^(4)^	2.33 [2.0–3.0]
Postoperative analgesia, n (%)	40 (95)
Paravertebral catheter	31 (77)
Epidural catheter	9 (23)
None	2 (5)

Data available for 40 patients^(1)^, 20 patients^(2)^, 31 patients^(3)^, and 35 patients^(4)^

### Main postoperative outcomes

Main postoperative outcomes are summarized in Table 3. Extubation was performed in the operative room or within the first 24 h after surgery in most patients (n = 35; 83%). Among those latter, 2 required NIV and 3 required reintubation due to hypercapnic respiratory failure (Additional file 3: Fig. S1). Seven patients (17%) could not be extubated within the first 24 h after surgery (Additional file 4: Fig. S2). Six of them were hypoxemic with ventilator-associated pneumonia and one had difficulty coughing. Eleven patients (26%) developed a postoperative pneumonia, 8 of whom were mechanically ventilated, and 5 patients (12%) developed a postoperative ARDS. Two patients had a tracheotomy. The ICU and hospital lengths of stay were 6.5 days [4.0–9.0] and 11.5 days [9.0–16.0], respectively. Two patients (4%) died in the ICU.

**Table 3:** Main postoperative outcomes

Variable	Value
Ventilatory support	
Duration of postoperative mechanical ventilation (days), median [IQR 25–75]	0 [0–1.0]
Early extubation	35 (83)
NIV or nasal high flow nasal oxygen (HFNO)	2
Reintubation	3
Late extubation (no extubation possible within the first 24 h)	7 (17)
Postoperative organ failure, n (%)	
Postoperative respiratory failure	12 (28)
Postoperative hemodynamic failure*	6 (14)
Postoperative renal failure	1 (2)
Postoperative infectious events**	
Postoperative pneumonia, n (%)	11 (26)
Ventilator associated pneumonia	8
Surgical site infection, n (%)	0
ICU stay	
Death	2 (4)
Length of stay (days), median [IQR 25–75]	6.5 [4.0–9.0]
Hospital stay	
Death	0
Length of stay (days), median [IQR 25–75]	11.5 [9.0–16.0]

NIV, Non-invasive ventilation

*One patient had an acute coronary syndrome (ST+)

**The numbers of postoperative pneumonia are 3 and 8 in non-mechanically ventilated and mechanically ventilated patients, respectively

### Factors associated with postoperative complications

**Fig. 3:**
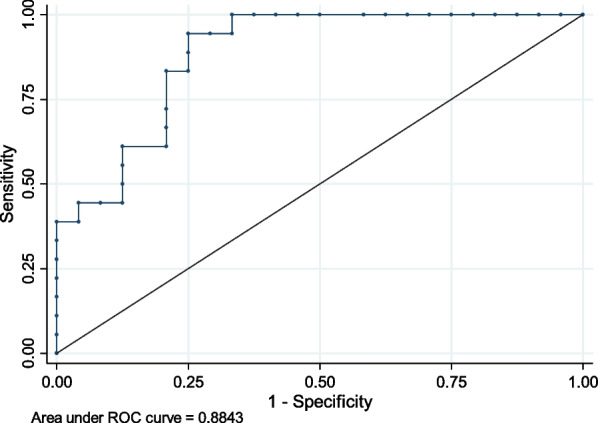
Diagnostic performance of TTSS and SAPSII without age for predicting postoperative complications

A postoperative complication occurred in 18 patients (43%). Factors associated with the occurrence of postoperative complications are detailed in Table 4. Comorbidities (diabetes and ischemic heart disease), high TTSS, bilateral fractures, other chest trauma (clavicle, sternum, scapula or vertebrae fractures), initial clinical severity (initial respiratory failure, tracheal intubation with mechanical ventilation, SAPSII score) and the need for intraoperative transfusion (transfusion, number of units) were associated with the occurrence of postoperative complications. In multivariable analysis, the TTSS (OR = 1.89; IC95% 1.12–3.17; *p* = 0.016) and the SAPSII without age (OR = 1.17; IC95% 1.02–1.34; *p* = 0.024) were independently associated with the occurrence of postoperative complications, with good discrimination (area under the ROC curve 0.88) and calibration (Hosmer Lemeshow test 0.696) (Fig. 3). The ISS score was not associated with the occurrence of postoperative complications.

**Table 4:** Factors associated with postoperative complications

Variable	No postoperative complication, n = 24	Postoperative complication, n = 18	OR [CI 95%]	*p*
Demographics				
Age (years), median IQR [25–75]	52.4 [37.4–73.8]	69.01 [54.8–79.6]		0.09
Sex (M, F)	18.6	10.8	0.42 [0.11–1.62]	0.19
Charlson score, median IQR [25–75]	0 [0–1]	1 [0–2]		0.03
Active smoking, n (%)	12 (50)	5 (28)	0.38 [0.099–1.49]	0.15
Characteristics of trauma				
Road traffic accidents, n (%)	12 (50)	8 (44)	0.80 [0.23–2.77]	0.72
Domestic fall, n (%)	9 (38)	8 (44)	1.33 [0.38–4.71]	0.65
Time from trauma to ICU referral (days), median [IQR 25–75]	2 [1–6]	1.5 [0–3]		0.34
TTSS, median [IQR 25–75]	10 [8–11]	12 [11–14.75]		0.0005
Bilateral thoracic trauma, n (%)	2 (8)	8 (44)	8.80 [1.29–59.92]	0.007
Other chest fractures, n (%)	7 (29)	13 (72)	5.20 [1.15–23.53]	0.017
Number of broken ribs, median IQR [25–75]	6.5 [5–7.25]	8 [5.25–11.5]		0.086
Extra-thoracic trauma	10 (42)	7 (38)	0.89 [0.25–3.15]	0.86
Severity factors at initial management				
ISS, median IQR [25–75]	41.5 [16–29]	26 [16–27]		0.59
SAPSII, median IQR [25–75]	19 [15–26]	33 [30–38]		0.0001
SAPSII without age, median IQR [25–75]	10 [7–17.5]	20 [14–25]		0.001
Respiratory failure, n (%)*	3 (13)	9 (50)	7.00 [1.29–37.93]	0.008
Peroperative management				
Time from trauma to surgery (days), median [IQR 25–75]	4.5 [2–7.25]	3 [1–6]		0.24
Duration of surgery (minutes), median [IQR 25–75]	125 [120–150]	150 [120–180]		0.11
Transfusion of red blood cells, n (%)	1 (4)	5 (28)	8.85 [0.79–99.31]	0.03
Number of red blood cells, median IQR [25–75]	0 [0–0]	0 [0–2]		0.004
Vasopressor support, n (%)	4 (9)	7 (17)	2.86 [0.64–12.81]	0.15
Loco regional analgesia	24 (100)	16 (88)		0.09
Postoperative support and outcomes				
Mechanical ventilation duration (days), median IQR [25–75]	0 [0–0]	3.5 [0–8.0]		0.0004
ICU length of stay, median IQR [25–75]	5 [3.75–7]	9 [6.25–17.5]		0.0002
In-ICU mortality, n (%)	0 (0)	2 (11)		0.9
Hospital length of stay, median IQR [25–75]	10 [7–12.25]	17 [11.25–31.5]		0.0002

COPD, Chronic Obstructive Pulmonary Disease; TTSS, Thoracic Trauma Severity Score; SAPSII, Simplified Acute Physiology Score; ISS, Index Severity Score; ICU, Intensive Care Unit

*Respiratory failure: need for high flow nasal oxygen (HFNO), non-invasive ventilation (NIV), or intubation with invasive ventilation

### Long-term outcomes (planned post-hospitalization visits)

At one month, data were available for 37 patients (92%). All were alive, and 8 patients had chronic neurological pain assessed by DNA4 questionnaire. Treatment with pregabalin was started. At one year, data were available for 26 patients (65%) and all were alive.

## Discussion

In this observational retrospective cohort study, we describe the characteristics, the management and the outcomes of 42 patients referred to the ICU of a referral thoracic center for surgical rib fracture fixation after a severe chest trauma, using the titanium plate of the Stracos™ system.

Our cohort is original, as it included different patients than those described in previous randomized controlled trials [14–16] (Additional file 1: Table S3). This was a selected population of patients transferred from trauma center with only isolated chest trauma. Our patients had more domestic accidents, were older, and had no respiratory failure in two third of the cases.

Postoperative complications occurred in 18 patients (43%) including two ICU deaths. This high rate of postoperative complications may question the role of surgery in such a selected population, particularly in old patients with severe trauma. The formalized experts French guidelines published in 2015 and 2017 recommend surgical rib fixation «in mechanically ventilated patients if the respiratory condition does not allow weaning from mechanical ventilation within 36 hours of admission», on the basis of three small size randomized clinical trials, two of which no longer reflect current practices [14–16].

These results are confirmed by more recent retrospective series, where operative rib fixation has the potential to reduce ventilator days and ICU stay in selected patients with severe traumatic flail chest requiring mechanical ventilation [22].

The radiological severity (flail chest) of the chest trauma may out weight the clinical and respiratory status of the patient in the surgical decision. However, the benefit of SRFF in non-ventilated patients remains to be determined. In a recent case–control series involving non-mechanically ventilated patients with flail chest, the patients who were treated non-surgically had better outcomes than their counterparts, with shorter duration of mechanical ventilation, lower rate of post-operative pneumonia and shorter ICU and hospital lengths of stay [21]. One of the main expected benefits of surgery is to decrease the duration of mechanical ventilation and reduce the associated morbidity. By restoring parietal rigidity, better wall mobility should be achieved, facilitating the restoration of proper ventilation. However, this hypothesis has not been confirmed in any controlled trial, and large variations in the duration of mechanical ventilation have been reported [15–17]. In the most recent series by Marasco et al. [15] the duration of mechanical ventilation was similar in the surgical group (6.3 days ± 3.4), as compared with the conservative group (7.5 days ± 5.4). Several hypotheses may explain the lack of benefits of surgery on the duration of mechanical ventilation. First, the chest wall mobility restoration and the expected better pulmonary compliance could be insufficient, due to the rigidity of the material used. In our series, the titanium claw plates were used. These clips simplify the fixation of screwless plates but there is no evidence that they may reduce the risk of intercostal neurovascular damage. Second, the surgical procedure itself may be more deleterious than beneficial. The complications associated with selective intubation [23, 24], one-lung ventilation, lateral decubitus positionning [25, 26] and the consequence of perioperative fluid administration and transfusion must be weighed against the expected advantages of surgery. A muscle-sparing approach, less invasive than posterolateral thoracotomy, could be considered as suggested in a recent series [27]. Data from the literature on other postoperative respiratory outcomes are unclear. The incidence of ARDS is not known but it increases with the number of fractured ribs [28]. Its incidence has decreased since the introduction of protective ventilation [29]. Postoperative pneumonia is not clearly defined in the literature and its incidence varies from 10 to 48% [15, 17]. In our series, only the episodes with high clinical suspicion of pneumonia and microbiological documentation were considered, accounting for an overall incidence of postoperative pneumonia of 26%. A reduction in the length of stay is also expected with the surgical treatment, but results are conflicting [15, 16, 20, 30]. The most recent randomized controlled study conducted in the United Kingdom with the length of stay as the primary end point, reported a significantly shorter length of stay in surgical patients, as compared with their counterparts (14.5 days vs. 30 days) [30]. The length of stay was longer than in other series [20, 21, 31, 32].

The optimal time for surgery is also important, as delayed surgery may result in pathological bone consolidation. Recent case–control studies [18–20] have reported a shorter duration of post-operative mechanical ventilation in patients operated within the first 4–5 days after trauma. In our series, surgery was performed after a median time of 4 days.

The three existing randomized controlled trials do not allow determining whether a surgical approach can benefit in non-intubated and old patients, as in our series. There are several retrospective series with similar population. Farquhar et al. [21] reported an increased length of stay in operated patients (7.4 ± 6.7 days), as compared with non-operated patients (3.7 ± 6.0 days), but surgery was performed a week after trauma. Wijffels et al. [31] reported a lower rate of postoperative pneumonia and a shorter hospital length of stay in operated patients, at a price of a higher number of surgery-related complications. Regarding geriatric population, Chen Zhu et al. [20] reported a decreased ICU and hospital lengths of stay in operated patients, as compared with non-operated patients (3 days vs. 7 days). Ali-Osman et al. [32] suggested an improved pulmonary function among operated patients despite an increased hospital length of stay.

Altogether, the benefits of surgery seem to exist when performed early after trauma in young patients with respiratory failure despite effective analgesia. In patients without respiratory failure or in older patients, surgical treatment is probably more controversial.

### Limitation of the study

The main limitations of our study are related to its retrospective and single center nature, as well as its sample size. The initial medical management of patients was not protocolized, since they were taken care of in a trauma center before being referred to our hospital. Our population was selected for receiving SRFF in an expert center, which makes the results less generalizable. The impact of the surgery on long-term respiratory function and chronic pain was not evaluated.

To summarize, we describe a cohort of 42 thoracic trauma patients with flail chest who received SRFF with titanium plates in an expert thoracic center. The TTSS and SAPSII score without age were independently associated with postoperative complications that occurred in 43% of cases. Conversely, the ISS score was not associated with such complications.

Further trials will help to provide answers about the benefit of the surgery in non-mechanically ventilated patients with isolated chest trauma.

## Supplementary Information

**Additional file 1: Table S1.** Thoracic Trauma Severity Score. **Table S2.** Summary of characteristics and outcomes of patients treated with rib fixation in different recent series. **Table S3.** Descriptive table of associated extra thoracic lesions.

**Additional file 2:** Protocol for enhanced rehabilitation after surgery.

**Additional file 3: Figure S1.** Post-operative outcomes in early extubated patients (first 24 hours after surgery)

**Additional file 4: Figure S2.** Post-operative outcomes in late extubated patients (beyond the first day of surgery).

## Data Availability

Consultation by the editorial board or interested researchers may be considered, subject to prior determination of the terms and conditions of such consultation and in respect for compliance with the applicable regulations.
