# Supplementary material for: Surgical and perioperative management of flail chest with titanium plates: a French cohort series from a thoracic referral center
Source: J Cardiothorac Surg. 2023 Jan 18;18:37. doi: 10.1186/s13019-023-02121-8 (PMC9850677; doi:10.1186/s13019-023-02121-8)
Supplement: Supplementary file 1 — Additional file 1: Table S1. Thoracic Trauma Severity Score. Table S2. Summary of characteristics and outcomes of patients treated with rib fixation in different recent series. Table S3. Descriptive table of associated extra thoracic lesions. [file 13019_2023_2121_MOESM1_ESM.pdf]

Table S1. Thoracic Trauma Severity Score

PaO2/FiO2 (mmHg)	Rib fracture	Contusion	Pleural involvement	Age (years)	Points
>400	0	None	None	<30	0
300-400	1-3	1 lobe	Pneumothorax	30-41	1
200-300	4-6<br>Unilateral	1 lobe bilateral<br>or 2 lobes unilateral	Unilateral hemothorax<br>Or hemopneumothorax	42-54	2
150-200	>3<br>Bilateral	< 2 lobes bilateral	Bilateral hemothorax<br>Or hemopneumothorax	55-70	3
<150	Flail chest	≥ 2 lobes bilateral	Tension pneumothorax	>70	5

Table S2. Descriptive table of associated extra thoracic lesions.

Localisation	Injury description	Number of patients concern
Head and neck	• Non-displaced fracture of C2<br>• Minor subarachnoidal hemorrhage<br>• Minor intraparenchymal haemorrhage<br>• Traumatic injury of the Petrous part of the Temproal Bone	2
Face	• Traumatic orbital apex syndrome<br>• Nose fracture<br>• Complexe facial trauma	4 (2 of which requiring surgery)
Extremities	• Upper limb fracture (Humeral, Forearm)<br>• Pelvic frature<br>• Lower limb fracture (femur, leg, ankle)	5 (3 of which requiring surgery)
Abdomen	• Spleen laceration<br>• Spleen fracture (needing embolisation)<br>• Subsapsular renal hematoma	3
Spine	• Fracture in the lumbar and thoracic vertebrae	3 (2 of which requiring surgical or radiological procedures)

Number of patients with associated extrathoracic trauma n = 17

Table S3. Summary of characteristics and outcomes of patients treated with rib fixation in different recent series

	Tanaka et al	Grenetzy et al	Marasco et al	Féray et al
Year of publication	2002	2005	2013	2019
Country	Japan	Egypt	Australia	France
Study type	Randomized controlled trial	Randomized controlled trial	Randomized controlled trial	Retrospective observational cohort
Number of operated/non operated patients	18/37	20/40	23/46	42/0
Age (years)	43 ± 12	40.5 ± 8.2	57.8 ± 17.1	59.7 ± 19.3
Sex ratio M/F	12/6	17/3	20/3	28/14
COPD, n (%)<br>Asthma, n (%)<br>Smoker, n (%)	NA	NA	2 (9)<br>3 (13)<br>8 (35)	4 (9)<br>2 (5)<br>17 (40)
Road traffic accident, n (%)			19 (82)	20 (48)
Number of broken ribs	8.2 ± 3	4.4	11.0 ± 3.1	7.3 ± 2.8
Patients on mechanical ventilation before surgery, n (%)	18 (100)	NA	23 (100)	6 (14)
Time from trauma to surgery, days	1 week	24-36 hours	In the 48 hours after randomization	4.0 days [2-6]
Number of days on mechanical ventilation after surgery	2.5 ± 3.2	2	NA	4.9 ± 11.5
Length of stay, days<br>ICU<br>Hospital	 16.5 ± 7.4<br>NA	 9.6<br>11.7	 13.5 [9.9-15.8]<br>20 [18-28]	 6.50 [4.0-9.0]<br>11.5 [9.0-16.0]
Postoperative pneumonia, n (%)	4 (22)	2 (10)	11 (48)	19 (45)
Tracheotomy, n (%)	3	NA	9 (39)	2 (5)
Mortality, n (%)	0 (0)	2 (10)	0 (0)	2 (5)

Abbreviations: COPD, Chronic Obstructive Pulmonary Disease; ICU, Intensive Care Unit; NA, Not Available
