# Supplementary material for: Surgical and perioperative management of flail chest with titanium plates: a French cohort series from a thoracic referral center
Source: J Cardiothorac Surg. 2023 Jan 18;18:37. doi: 10.1186/s13019-023-02121-8 (PMC9850677; doi:10.1186/s13019-023-02121-8)
Supplement: Supplementary file 2 — Additional file 2: Protocol for enhanced rehabilitation after surgery. [file 13019_2023_2121_MOESM2_ESM.pdf]

PROTOCOL FOR ENHANCED REHABILITATION AFTER THORACIC SURGERY

Preoperative period

Hygienic and dietary rules

Due to the emergency of the surgery, nutritional measures, physical exercise and cardiovascular preparation could not be implemented.

The time frame was also too short for smoking cessation, but nicotinic substitution was prescribed to all smokers

Postoperative period

All patients were admitted to intensive care units for a minimum of 24 hours.

Pain Control

Paravertebral catheter: Patient control analgesia (Ropivacaine 2 mg / ml) with continuous flow 10 ml/h + bolus of 5 ml every 20 minutes

Thoracic Epidural catheter: Patient control epidural analgesia (Ropivacaine 2 mg / ml + Sufentanil 0.5 µg/ml) continuous flow 3 to 5 ml/h + bolus 3 to 5 ml every 20 minutes.

In all cases association to a multimodal analgesia, respecting the possible contra-indications:

- Acetaminophen 1g x4/j oral
- Nefopam 20 mg x4/j oral
- NSAIDs (Ketoprofen) 50 mg x3/j oral for 48h
- oral morphine sulfate 10 mg x6/j if EVA > 3/10 despite other pain killers

Effectiveness of analgesia assessed by measuring the VAS score at least every 4 hours, targeting a VAS ≤ 3.

Chest tube management:

Common management with thoracic surgery team:

Early cessation of suction / removing of chest tube as soon as possible (lung reattached to the wall, fluid output < 500 ml/24h).

A digital autonomous drainage system was used in order to facilitate mobilization.

Daily chest x-ray in bed at least as long as the chest tube is in place.

Urinary catheter management:

No systematic urinary catheterization except when an epidural was in place.

Bladder scan if no resumption of spontaneous urination, indication for urine drainage if > 400 ml.

Early mobilization and respiratory physical therapy

Systematically put in the chair on Day 0.

First walk at D0 if the patient is arriving in intensive care unit before 8 pm, otherwise on the morning of D1.

Requirement for the first standing:

- Physiotherapist should be present if possible

- Two caregivers minimum

- Two-step procedure: bedside then standing

- Blood pressure and oxygen saturation monitoring

If the first walk is well tolerated:

- Mobilization several times per day with a rollator
- Daily respiratory physiotherapy: active cycle of breathing technique with thoracic expansion exercises, volume-incentive spirometer, positive expiratory pressure devices.

Early oral intake

Systematic swallowing test, performed in the first 2 hours after extubation

Resume oral feeding as soon as possible

Thromboprophylaxy

Enoxaparin: 4000 UI per day for every patients or 6000 UI per day if BMI > 30kg/m2.
