# Supplementary material for: Surgical and perioperative management of flail chest with titanium plates: a French cohort series from a thoracic referral center
Source: J Cardiothorac Surg. 2023 Jan 18;18:37. doi: 10.1186/s13019-023-02121-8 (PMC9850677; doi:10.1186/s13019-023-02121-8)
Supplement: Supplementary file 3 — Additional file 3: Figure S1. Post-operative outcomes in early extubated patients (first 24 hours after surgery) [file 13019_2023_2121_MOESM3_ESM.pdf]

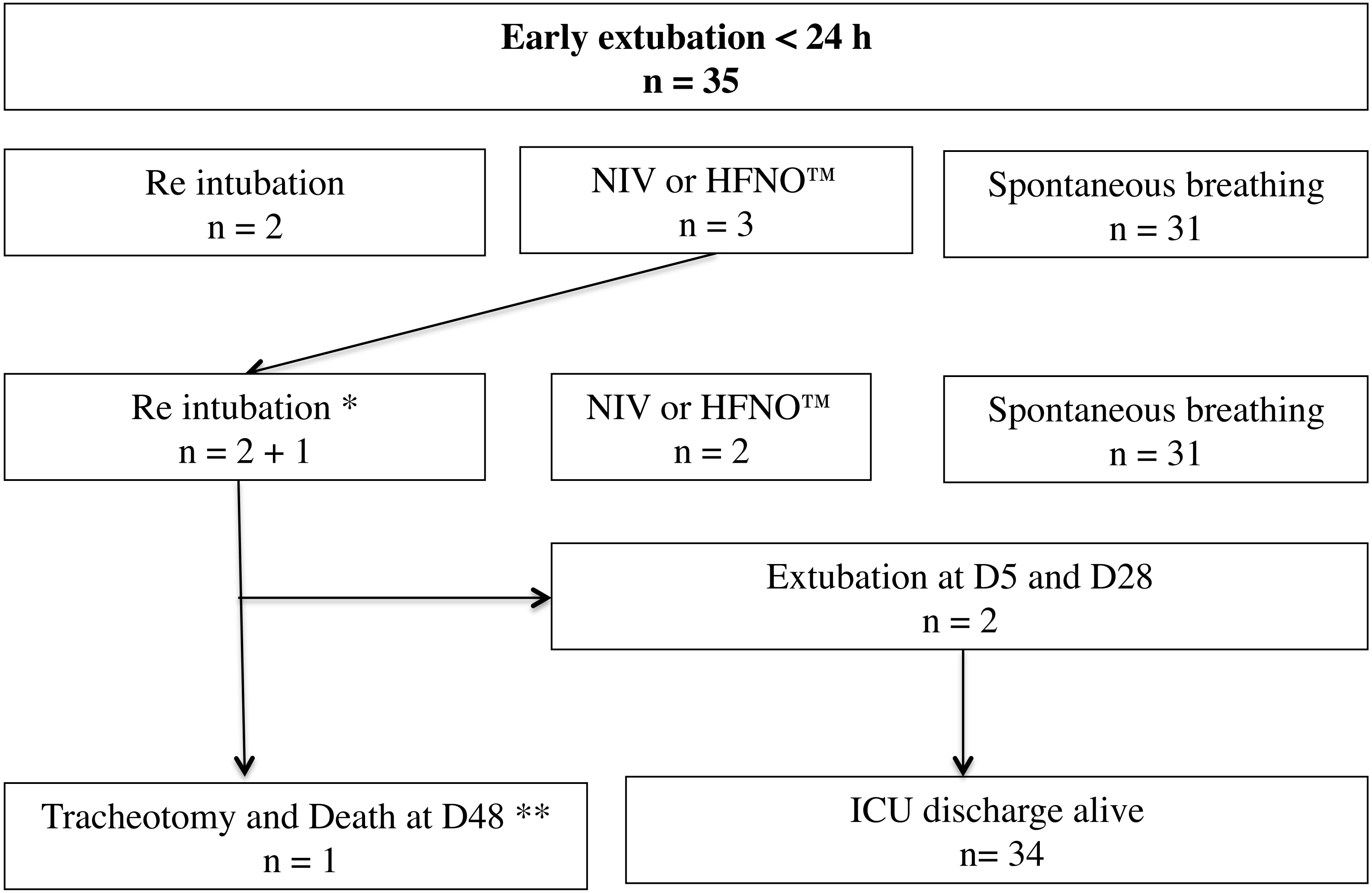

NIV : non invasive ventilation, High flow nasal oxygen therapy (HFNO)

** Two patients were reintubated within few hours of extubation due to hypercapnic respiratory distress and one after 48 hours of NIV*

*** The death occurred 48 days after surgery, after a collegial and multidisciplinary decision to withdraw therapies.*
