# Supplementary material for: Surgical and perioperative management of flail chest with titanium plates: a French cohort series from a thoracic referral center
Source: J Cardiothorac Surg. 2023 Jan 18;18:37. doi: 10.1186/s13019-023-02121-8 (PMC9850677; doi:10.1186/s13019-023-02121-8)
Supplement: Supplementary file 4 — Additional file 4: Figure S2. Post-operative outcomes in late extubated patients (beyond the first day of surgery). [file 13019_2023_2121_MOESM4_ESM.pdf]

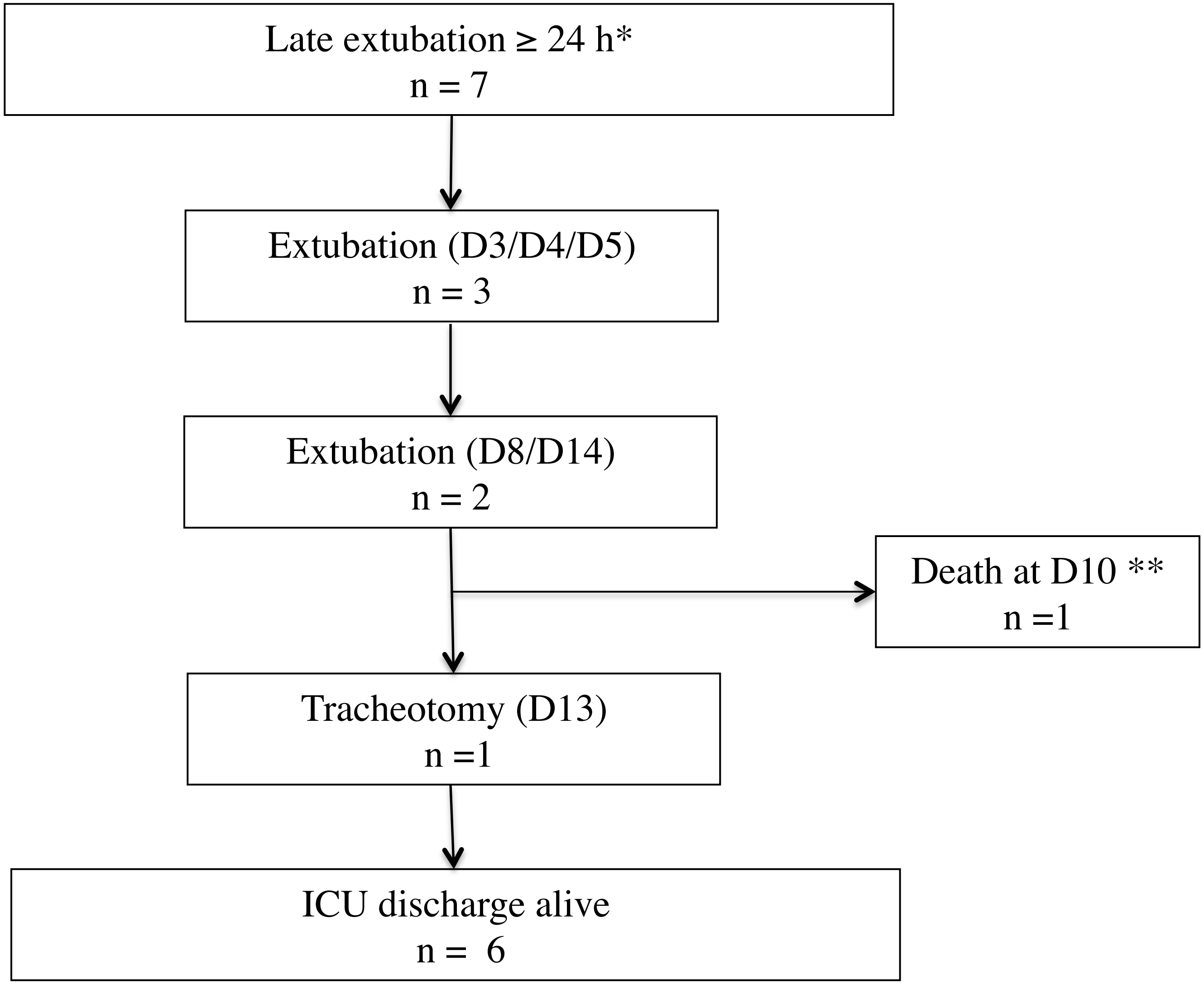

**The reasons for delayed extubation were hypoxemia related to ventilator-associated pneumonia (n=6) and poor ventilator mechanics and cough difficulty (n=1).*
***A cardiac arrest occurred 10 days after surgery, after 48 hours of extubation. The cause of death was unknown*
